# Evaluating mammographic density′s contribution to improve a breast cancer risk model with questionnaire-based and polygenic factors

**DOI:** 10.1038/s41523-025-00813-z

**Published:** 2025-10-01

**Authors:** Charlotta V. Mulder, Xin Yang, Yon Ho Jee, Christopher G. Scott, Chi Gao, Yu Cao, Amber N. Hurson, Mikael Eriksson, Celine M. Vachon, Per Hall, Antonis C. Antoniou, Peter Kraft, Gretchen L. Gierach, Montserrat Garcia-Closas, Parichoy Pal Choudhury

**Affiliations:** 1 Division of Cancer Epidemiology and Genetics, National Cancer Institute, Bethesda, MD USA; 2 Department of Molecular Pathology, The Netherlands Cancer Institute, Amsterdam, Netherlands; 3 Department of Public Health and Primary Care, University of Cambridge, Cambridge, UK; 4 Department of Epidemiology, Harvard TH Chan School of Public Health, Boston, USA; 5 Department of Health Sciences Research, Mayo Clinic, Rochester, USA; 6 American Cancer Society, Atlanta, USA; 7 Department of Medical Epidemiology and Biostatistics, Karolinska Institutet, Campus Solna, Sweden; 8 Cancer Epidemiology and Prevention unit, Institute of Cancer Research, London, UK; 9 Imperial College London, London, UK

**Keywords:** Breast cancer, Cancer epidemiology

## Abstract

Incorporation of mammographic density into breast cancer risk models may improve risk stratification for tailored screening and prevention. We evaluated the added value of Breast Imaging Reporting and Data System (BI-RADS) breast density to a validated model combining questionnaire-based risk factors and a 313-variant polygenic risk score (PRS), using the Individualized Coherent Absolute Risk Estimator (iCARE) tool for risk model building and validation. Calibration and discrimination were assessed in three prospective cohorts of European-ancestry women (1468 cases, 19,104 controls): US-based Nurses’ Health Study (NHS I and II) and Mayo Mammography Health Study (MMHS); and Sweden-based Karolinska Mammography Project for Risk Prediction of Breast Cancer (KARMA) study. Analyses were stratified by age (<50, ≥50 years). Adding density modestly improved discrimination: among younger women, AUC increased from 65.6% (95% CI: 61.9–69.3%) to 67.0% (95% CI: 63.5–70.6%); among older women, from 65.5% (95% CI: 63.8–67.2%) to 66.1% (95% CI 64.4–67.8%). Among US women aged 50–70 years, 18.4% were identified at ≥3% 5-year risk with density included, capturing 42.4% of future cases; 7.9% were reclassified, identifying 2.8% more future cases. In Sweden, 10.3% were identified at elevated risk, capturing 29.4% of cases, with 5.3% reclassified and 4.4% more cases identified. Integrating density with established risk factors and PRS may enhance breast cancer risk stratification among European-ancestry women, supporting its potential for clinical utility.

## Introduction

Clinical application of risk-stratified breast cancer prevention strategies in the population requires the development and robust prospective validation of flexible and comprehensive models for absolute risk prediction to provide accurate individualized risk estimates, in particular for women at high risk for whom such applications have the greatest potential impact^1,2^. Several risk models have been developed incorporating different sets of risk factors and targeting different clinical scenarios^3,4^; however, further work is needed to demonstrate whether improvements in risk stratification of current models may be achieved by incorporating additional risk factors, ultimately enhancing our ability to identify women at the extremes of risk distribution.

In our previous work, we built and validated a literature-based 5-year breast cancer prediction model incorporating reproductive, lifestyle and behavioral factors, family history and the recently developed PRS composed of 313 common variants^5^ with the Individualized Coherent Absolute Risk Estimator (iCARE) software tool^6^. This tool provides a flexible framework for absolute risk model development, aggregating information on risk-factor associations, population-based age-specific disease incidence rates and competing mortality rates and the risk-factor distributions from multiple data sources, and further implements standardized model validation methods. The model with questionnaire-based risk factors and PRS showed good calibration in multiple populations of European-ancestry women^7^. Moreover, we also predicted that adding mammographic breast density to this model could further improve risk stratification^8^.

Since the discovery by Wolfe in 1976^9^, density has been consistently shown to be a strong risk factor for breast cancer. The radio-opaque structures on a mammogram indicate stromal and epithelial tissue, while the radiolucent area indicates adipose tissue^10^. Currently, the most widely used clinical system to classify density is the Breast Imaging-Reporting and Data System (BI-RADS), where density is visually assessed by a radiologist and categorized into four levels: almost entirely fatty, scattered areas of fibro-glandular density, heterogeneously dense, or extremely dense^11^. Population-wide studies have demonstrated that approximately 50% of the US female population aged 40–74 years have heterogeneously or extremely dense breasts, with extremely dense breasts conferring a two- to four-fold higher relative risk compared to almost entirely fatty breasts^12–15^.

Breast density has been incorporated into established risk models like the Gail model, the Breast Cancer Surveillance Consortium (BCSC) model, the Tyrer-Cuzick (IBIS) model, Rosner-Colditz model and the Breast and Ovarian Analysis of Disease Incidence and Carrier Estimation Algorithm (BOADICEA)^16–23^. The BCSC, Tyrer-Cuzick and BOADICEA models also incorporate density into their clinical risk calculator tools^1–3^. A robust evaluation of the added value of mammographic breast density to a model with comprehensive information on questionnaire-based risk factors and the most recent polygenic risk scores are needed to determine its effectiveness in improving risk stratification of models.

In our current work, we validate the fully integrated model with questionnaire-based risk factors, the 313-SNP PRS and density for calibration and discrimination in three prospective cohorts of European-ancestry women (two from the US and one from Sweden), totaling 1468 cases and 19,104 controls. Our risk projection and reclassification calculations in the populations of European-ancestry women aged 50–70 years from the US and Sweden show the improvements in risk stratification attainable by incorporating density to the current literature-based model with questionnaire-based risk factors and PRS.

## Results

### Model validation results

**Fig. 1:**
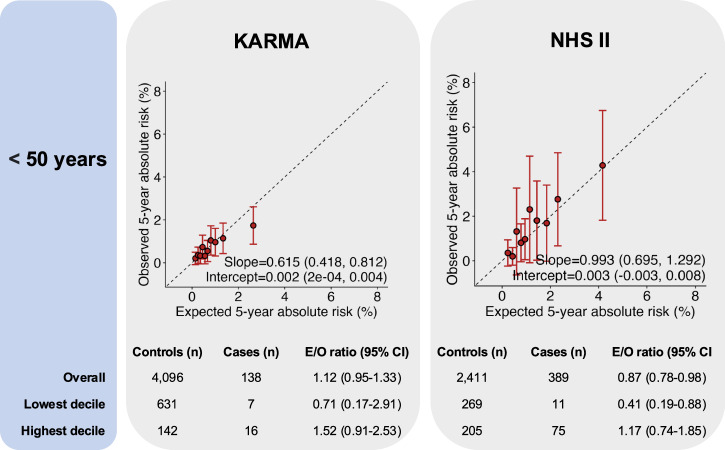
Calibration and discrimination of 5-year risk predictions of breast cancer for women aged younger than 50 years. Calibration and discrimination of 5-year risk predictions of breast cancer for women aged younger than 50 years in the nested case-control sample of NHS II and KARMA with risk categories based on deciles of predicted 5-year absolute risk. Validation results are shown for the extended iCARE model that incorporates questionnaire-based risk factors with a PRS based on 313 common germline variants and density. Estimates and 95% CI of the calibration slope and intercept are reported based on a linear regression of the decile-specific observed proportion of cases within 5 years and the average of the predicted 5-year absolute risk. AUC area under the curve, *c*^2^*χ*^2^ goodness-of-fit test, CI confidence interval, E/O expected to observed number of cases, KARMA Karolinska Mammography Project for Risk Prediction of Breast Cancer, NHS II Nurses’ Health Study II, PRS polygenic risk score, QRF questionnaire-based risk factors.

For women younger than 50 years, the integrated model with questionnaire-based risk factors, PRS and density was generally well calibrated in terms of relative and absolute risk across the risk categories in NHS II and KARMA (Fig. 1 and Fig. S1A, B). In the meta-analysis across studies, the area under the curves (AUC) with and without density were 67.0% (63.5–70.6%) vs. 65.6% (61.9–69.3%), respectively (Fig. 3a and Table S6A).

**Fig. 2:**
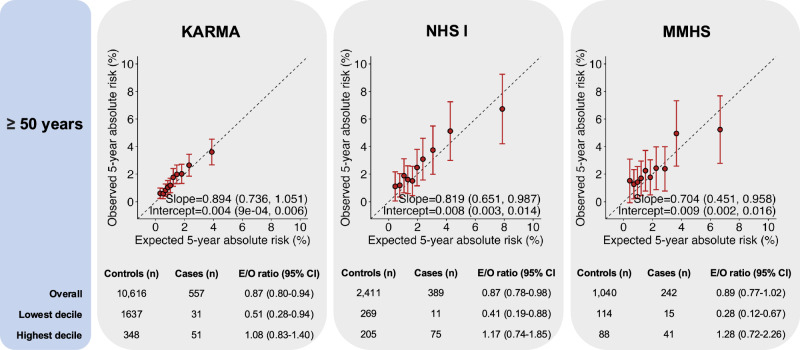
Calibration and discrimination of 5-year risk predictions of breast cancer for women aged 50 years and older. Calibration and discrimination of 5-year risk predictions of breast cancer for women aged 50 years and older in the nested case-control sample of KARMA, NHS I, and MMHS with risk categories based on deciles of predicted 5-year absolute risk. Validation results are shown for the extended iCARE model that incorporates questionnaire-based risk factors with a PRS based on 313 common germline variants and density. Estimates and 95% CI of the calibration slope and intercept are reported based on a linear regression of the decile-specific observed proportion of cases within 5 years and the average of the predicted 5-year absolute risk. AUC area under the curve, *c*^2^*χ*^2^ goodness-of-fit test, CI confidence interval, E/O expected to observed number of cases, KARMA Karolinska Mammography Project for Risk Prediction of Breast Cancer, MMHS Mayo Mammography Health Study, NHS I Nurses’ Health Study I, PRS polygenic risk score, QRF questionnaire-based risk factors.

**Fig. 3:**
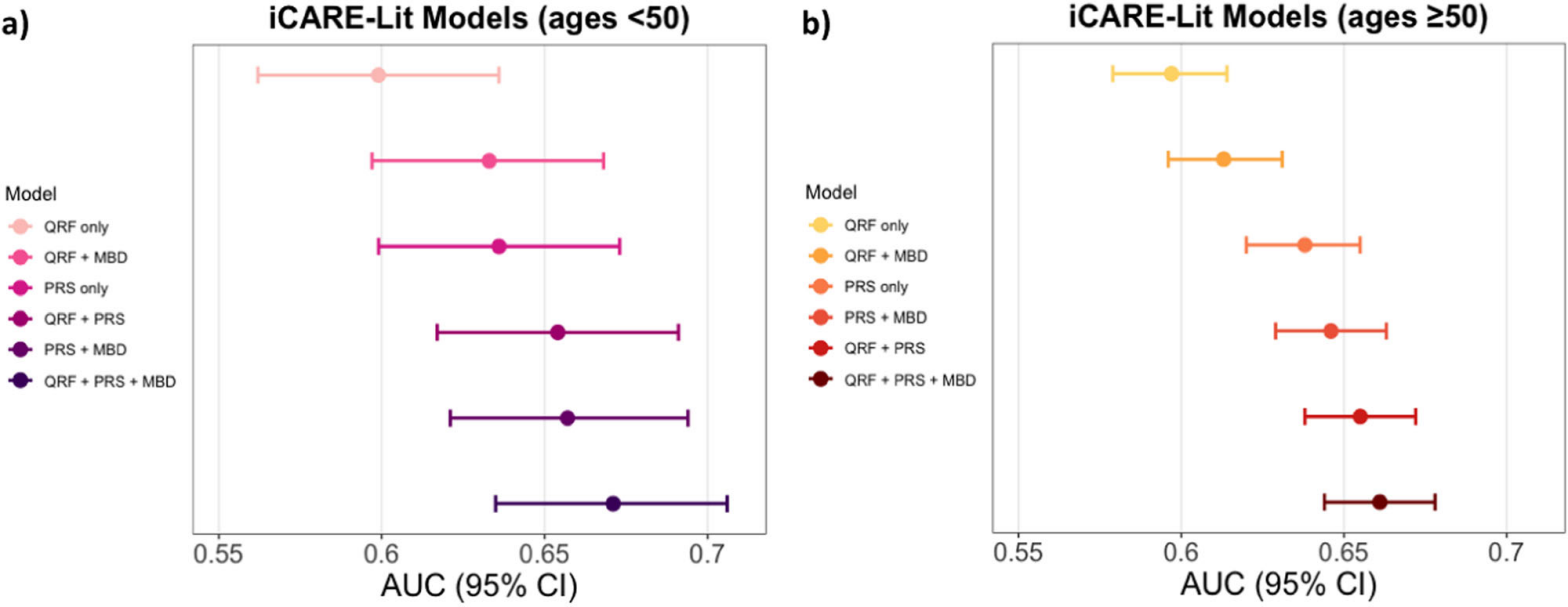
Risk discrimination is measured by the model area under the curve (AUC) of the iCARE-Lit models. Risk discrimination measured by the model area under the curve (AUC) of the iCARE-Lit models for **a** women younger than 50 and **b** 50 years or older based on a meta-analysis across studies for the risk-factor combinations: incorporates (i) questionnaire-based risk factors only, (ii) questionnaire-based risk factors with density, (iii) questionnaire-based risk factors with a PRS based on 313 common germline variants, (iv) the 313-variant PRS only, (v) the 313-variant PRS and density and (vi) the fully integrated model incorporating questionnaire-based risk factors, the 313-variant PRS and density. Colored dots were used to denote estimates, and colored horizontal lines denote the 95% confidence intervals. iCARE-Lit model based on literature review, MBD mammographic breast density, PRS polygenic risk score, QRF questionnaire-based risk factors.

For women 50 years or older, the integrated model showed some underestimation of overall risk in NHS I and KARMA (NHS I: E/O = 0.87 (0.78-0.98) and KARMA: E/O = 0.87 (0.80-0.94), Fig. 2 and Table S5). This was mainly noticeable in the lowest risk decile (KARMA: E/O = 0.51 (0.28-0.94), NHS I: E/O = 0.41 (0.19–0.88), MMHS: E/O = 0.28 (0.12–0.67), Fig. 2). For this age group, in the meta-analysis across studies, the AUCs with and without density were 66.1% (95% CI 64.4–67.8%) and 65.5% (95% CI: 63.8–67.2%) (Fig. 3b and Table S6B).

### Risk stratification and reclassification

**Fig. 4:**
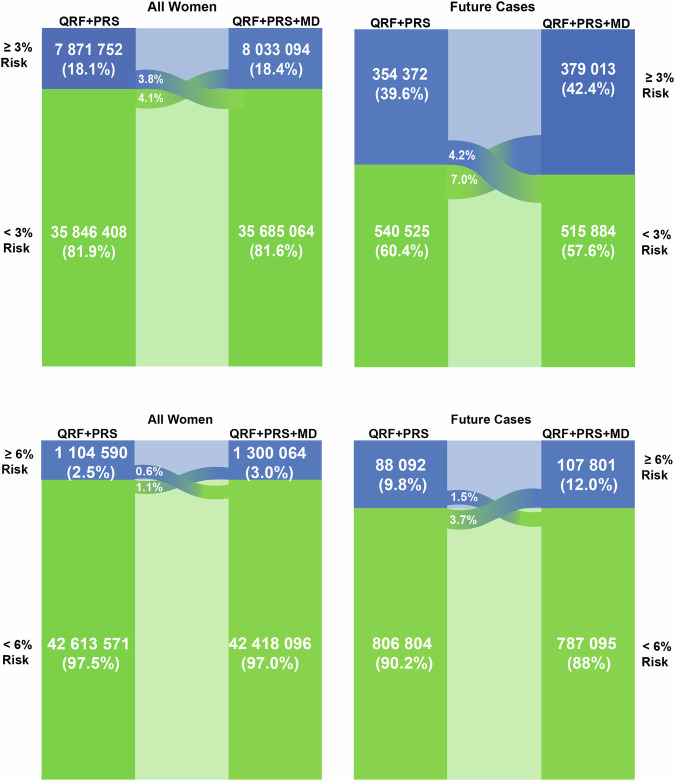
Reclassification of US women at two high-risk thresholds. Women of European-ancestry aged 50–70 years in the general population of the USA expected to be identified at high risk of breast cancer according to two risk thresholds, and the incident cases of breast cancer expected to occur in these groups within a 5-year interval, comparing the (i) questionnaire-based risk factor and PRS model with (ii) the fully integrated model with questionnaire-based risk factors, PRS and density. The expected number of women is calculated using 2020 (*N* = 43,718,160) population estimates from the US Census Bureau for the USA. The expected numbers of cases are estimated using the average predicted 5-year risk in the US population, calculated using the US breast cancer incidence rates and risk-factor distributions (Supplementary data). The 3% threshold is used by the US Preventive Services Task Force for recommending risk-reducing medications, and 6% is used by the WISDOM trial as a cutoff for very high risk^27,60^. AR absolute risk, BMI body mass index, MBD mammographic breast density, PRS polygenic risk score, QRF questionnaire-based risk factors, US United States.

The extended model with questionnaire-based risk factors, the 313-variant PRS and density identified 18.4% of the US non-Hispanic White population women 50–70 years old ≥3% predicted 5-year risk, the cut-off used for recommending risk-reducing medication in the United States (Fig. 4 and Table S7). This group includes 42.4% of predicted future cases (Fig. 4 and Table S7). The addition of density led to the reclassification of 7.9% of US non-Hispanic White women aged 50–70 years, with 4.1% moving from below the ≥3% predicted 5-year risk threshold to above and 3.8% moving in the opposite direction. This resulted in the identification of 2.8% of additional future cases (Fig. 4 and Table S8). At and above the 6% risk threshold, the high-risk cutoff in the WISDOM trial^24^, the fully integrated model identified 3.0% of the US non-Hispanic White population above 50–70 years. This group is expected to include 12.0% of future cases (Fig. 4 and Table S7). Among these women, 1.7% were reclassified, with 1.1% moving from below the ≥6% predicted 5-year risk threshold to above and 0.6% moving in the opposite direction. This led to the identification of 2.2% additional future cases (Table S8 and Fig. 4).

**Fig. 5:**
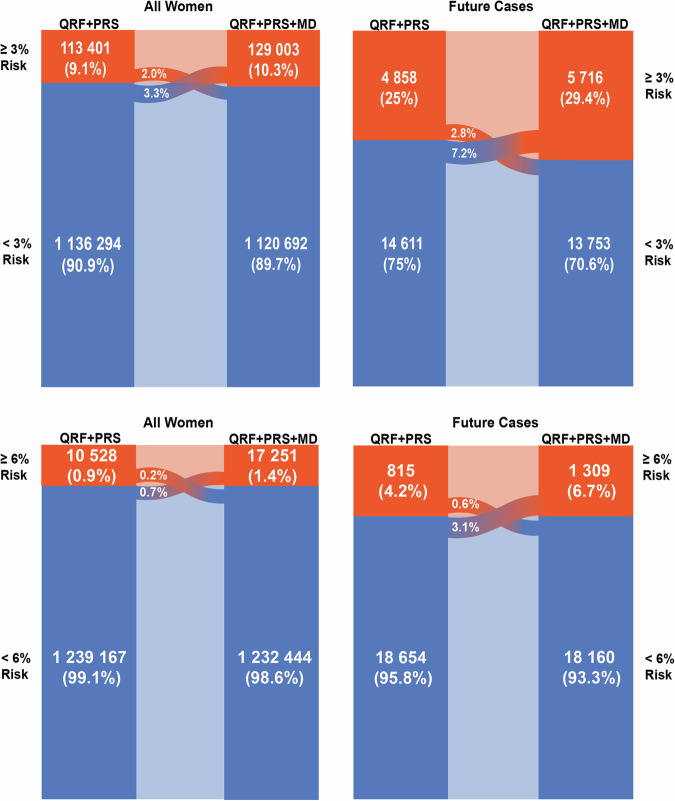
Reclassification of Swedish women at two high-risk thresholds. Women of European-ancestry aged 50–70 years in the general population of the USA expected to be identified at high risk of breast cancer according to two risk thresholds, and the incident cases of breast cancer expected to occur in these groups within a 5-year interval, comparing the (i) questionnaire-based risk factor and PRS model with (ii) the fully integrated model with questionnaire-based risk factors, PRS and density. The expected number of women is calculated using 2016 population estimates (*N* = 1,249,695) from Statistics Sweden for Sweden. The expected numbers of cases are estimated using the average predicted 5-year risk in the Swedish population, calculated using the SE breast cancer-incidence rates and risk-factor distributions (Supplementary Data). The 3% threshold is used by the US Preventive Services Task Force for recommending risk-reducing medications, and 6% is used by the WISDOM trial as a cutoff for very high risk^27,60^. AR absolute risk, BMI body mass index, MBD mammographic breast density, PRS polygenic risk score, QRF questionnaire-based risk factors, SE Sweden.

In the Swedish population, the integrated model identified 10.3% of women aged 50–70 years ≥3% predicted 5-year risk with 29.4% of future cases expected to occur in this group (Table S7 and Fig. 5). With the addition of density, 5.3% of women of European-ancestry were reclassified, with 3.3% from below to above and 2.0% in the opposite direction. This identified an additional 4.4% of future cases (Table S8 and Fig. 5). At and above the 6% risk threshold, the fully integrated model identified 1.4% of women of European-ancestry aged 50–70 in the Swedish population. 6.7% of future cases are expected to occur in this group (Table S7 and Fig. 5). With the addition of density, 0.9% of all women were reclassified above the ≥6% risk threshold, with 0.7% of women moving from below the threshold to above, and 0.2% moving in the opposite direction. This resulted in the identification of 2.5% additional future cases (Table S8 and Fig. 5).

## Discussion

We investigated the added value of incorporating breast density to the iCARE-Lit questionnaire-based risk factor and PRS model to predict 5-year absolute risk of breast cancer among women of European-ancestry. The addition of density to the model resulted in a modest improvement in risk stratification and reclassification. For instance, incorporating density identified an additional 2.8 and 4.4% of future cases at and above a 3% predicted 5-year risk threshold and an additional 2.2 and 2.5% of future cases at and above a 6% predicted 5-year risk threshold in populations of European-ancestry in the USA and Sweden, respectively. The model’s ability to stratify more women above and below clinically relevant risk thresholds would lead to more women being rightfully allocated to high and low-risk categories and, therefore, able to qualify for risk-reducing strategies.

Our model, with an AUC of 67.1% (Table S6A) in women younger than 50 years and 66.1% (Table S6B) in women 50 years or older, demonstrates modest risk discrimination. A key challenge in risk stratification based on models with modest risk discrimination is that a substantial percentage of breast cancer cases occur among women not classified to be at high risk. While the high-risk women could be targeted for certain interventions with inherent risks and benefits, the high proportion of cases outside the high-risk groups underscores the need for implementing broad, globally beneficial public health measures to reduce the population burden of breast cancer. These include lifestyle modifications such as maintaining a healthy BMI, increasing physical activity, and limiting alcohol intake, as advocated by the American Cancer Society and other public health organizations.

The implementation of such risk-stratified screening and prevention strategies of breast cancer in the population is a key goal of risk prediction efforts. Currently, two non-inferiority trials, WISDOM^24,25^ and MyPeBS^26^, are underway investigating the potential of risk-based personalized screening as a safe alternative to mammographic screening programs^27^. As present screening programs are based solely on age as an entry criterion; comprehensive risk models can pave the way for individualized risk-based screening strategies. For countries where density is collected routinely in the clinical setting, this would be a relatively simple addition to risk models. However, prior to implementing a model incorporating comprehensive questionnaire-based risk factors, PRS and mammographic breast density in clinical and screening practices, a formal clinical utility assessment should be carried out to assess effectiveness, cost-effectiveness, psychological, ethical and legal implications of an intervention.

Our model incorporating density showed some signs of miscalibration at the extremes of the distribution. Since the model is literature-based with the relative risks derived using a literature review, it is subject to structural assumptions about the underlying model. For women younger than 50 years, we obtained an age-adjusted relative risk for density from ref. ^19^, which did not account for the correlation of density with BMI^28^. Moreover, in both NHS and KARMA, mammographic density was measured using semi-automated software, CUMULUS and STRATUS, respectively, and converted to a four-level variable to approximate density. There might be some misclassification due to the conversion from percent density to the four-category visually assessed density. Conversely, in MMHS, the density categorization might have contributed to misspecification as it was visually assessed by radiologists, and although broadly accepted, the BI-RADS reporting system is subject to substantial intra- and inter-observer variability between radiologists, with Kappa values^29^ ranging between 0.4 and 0.7^4–7^. Several studies show a stronger relationship between breast cancer and percent density compared to BI-RADS density^30–33^. Incorporating automated quantitative mammographic features of mammographic images into risk models could address these limitations. Interestingly, ref. ^34^ found similar discriminatory accuracy between their model with automated or clinical density and ref. ^35^ found that adding both a clinical and automated volumetric density measure improved risk stratification.

Our validation was conducted using three relatively old prospective cohorts of women of European-ancestry in the USA and Sweden, which may not be fully representative of the general population. In particular, MMHS and KARMA cohorts consist of women participating in annual or biannual screening programs, respectively, and the NHS cohort is comprised of nurses, both of which are groups that tend to have higher educational attainment and healthier lifestyles compared with the general population. While this could influence the calibration of absolute risk, the calibration of relative risk estimates is less likely to be substantially affected. Importantly, since the primary application of our model is within breast cancer screening contexts, these cohorts remain highly relevant for our aims. Nevertheless, further validation is needed in more representative and contemporary populations, particularly those more closely aligned with the target settings of integrated healthcare systems, such as the CONNECT for Cancer Prevention Study^36^.

This paper has a notable strength in the breadth of the validation, as it assesses the model’s performance across three different studies with participants coming from two populations of European-ancestry women in the US and Sweden. Other validation studies either included a smaller number of participants or used combinations of a limited set of risk factors and polygenic risk scores (PRS) with fewer genetic variants (Table S9)^16,20,37–39^. Recently, the extended version of the BOADICEA model incorporating questionnaire-based risk factors, the 313-SNP PRS, density and rare moderate- and high-risk variants has also been externally validated in KARMA, similarly showing the importance of reclassification and risk stratification^37^. Other established models have also shown improvement of model discrimination after the incorporation of density^35,38,40–43^, however most have yet to be externally validated in fully independent cohorts. The independent external validation of risk models is critical before clinical applications; however, a potential barrier is the availability of large prospective cohorts with comprehensive genetic, mammography and risk-factor information. The Tyrer-Cuzick (v 7.02) model and the BCSC model (v2) with mammographic density have been validated in large independent cohorts; however, both these versions do not include genetic information^16,22^. In contrast to the three aforementioned validation studies, other established models like the Gail model have only been internally validated after the addition of breast density information^21,40^. Moreover, there is an increasing interest in using fully automated density measures using deep learning algorithms^44,45^. Using artificial intelligence on mammograms to aid risk prediction has the benefit of moving beyond density by also characterizing (micro)calcifications, focal masses, left-right asymmetry and a landscape of radiomic features^46–48^. However, many of these algorithms have yet to be independently validated in epidemiological and clinical studies.

In addition to incorporating additional mammographic features, future advancements in breast cancer risk prediction should aim to distinguish between less and more aggressive subtypes, in situ vs. invasive lesions, and ultimately, between cancers that are likely to be lethal and those that are not. Since subtypes of breast cancer differ in their risk-factor associations and preventive strategies are often tailored to specific subtypes (e.g., based on estrogen receptor status), subtype-specific risk models could improve the identification of women most likely to benefit from targeted interventions. In contrast, our current model—like many widely used tools—estimates the absolute risk of developing any breast cancer, irrespective of subtype or prognosis. While it does not predict breast cancer–specific mortality, it remains a valuable resource for guiding primary prevention and early detection. Although not all incident breast cancers are life-threatening, reducing overall incidence, including less aggressive forms, may help mitigate the burden of overdiagnosis, overtreatment, and associated morbidity. Ultimately, while models predicting the risk of lethal disease represent a critical future direction^49^, incidence-based models are currently more validated and actionable, and thus continue to play an essential role in risk-stratified screening and prevention.

To summarize, the incorporation of density to questionnaire-based risk factors and PRS results in modest improvements in the identification of European-ancestry women at elevated breast cancer risk. Additional prospective validation in diverse populations, in particular of non-European-ancestry women, are needed to ensure equitable clinical application.

## Methods

### Study populations

Model validation analyses were performed in three case-control samples nested in prospective cohort studies of European-ancestry women: the US-based Nurses’ Health Study (NHS I and II) and Mayo Mammography Health Study (MMHS), and the Sweden-based Karolinska Mammography Project for Risk Prediction of Breast Cancer (KARMA) study. In total, analyses were carried out in 1468 cases and 19,104 controls and were done separately for women younger (NHS II, KARMA; 280 cases, 5037 controls) and older than 50 years (NHS I, MMHS, KARMA; 1188 cases, 14,067 controls). Women with a prior history of breast and other cancers, except for nonmelanoma skin cancer, were excluded from the study. Women consented to the use of their genetic material, mammogram with density and completed a risk-factor questionnaire. For women with multiple mammograms or multiple questionnaires, the data closest to the DNA collection were used. In MMHS, density was obtained from routine clinical examination by attending radiologists. All four mammogram views (craniocaudal and mediolateral oblique for ipsilateral and contralateral sides) contribute to the assessment of density^50^. In both NHS and KARMA, mammographic density was measured using semi-automated software, Cumulus^51^ and STRATUS^52^, respectively, and converted to a four-level variable to approximate density categories following BI-RADS^53^. With Cumulus software, an area-based measure of mammographic density is estimated with user-defined thresholds to define dense tissue, and percent density (i.e., dense tissue area/total breast area) was categorized using the thresholds <10%, 10–24% 25–49%, and >50%^54,55^. With STRATUS, an area-based measure of mammographic density is estimated using a machine learning method. Thereafter, the percentage of MD is calculated as the ratio of dense tissue to the total area. This measure was then categorized to approximate BI-RADS categories using the thresholds <2%, 2–8%, 8–49%, and >49%^56^. Characteristics of the prospective cohort studies used for model validation and the distribution of risk factors is provided in the supplementary material (Tables S1, S2, respectively).

Breast cancer outcome was ascertained through linkage to SEER registries, state tumor registries, and pathology databases. A woman was considered a case when she developed incident primary breast cancer, either in situ or invasive, during the follow-up period. The follow-up for all subjects began one year after study entry. This was designed to reduce the impact of prevalent cancers, those already present at the time of screening, on the validation analyses. Follow-up ended at the last record of cancer registry linkage or 5 years, whichever came first.

### Risk model development

We used iCARE^6^ to build a model for the 5-year absolute risk of developing breast cancer, integrating questionnaire-based risk factors, PRS and density separately for women younger and older than 50 years. iCARE implements a flexible approach of estimating absolute risk by combining information on relative risks of the risk factors, age-specific incidence and competing mortality rates from multiple data sources, and an individual-level reference dataset of risk factors representing the underlying population. Age was used as a timescale for disease incidence modeling. The conditional relationship between age-specific incidence rates and the risk factors were assumed to follow a Cox proportional hazards model.

The questionnaire-based risk factors included were ages at menarche, first birth and menopause, parity, height, BMI, alcohol intake, family history (i.e., presence/absence of breast cancer in at least one first-degree relative), history of benign breast disease, oral contraceptive use, menopausal hormone therapy (MHT) use, and current MHT type. Our previous works^7,8^ describe the integration of questionnaire-based risk factors and the 313-variant PRS^5^, and here we further extend the model incorporating density. The relative risks for the questionnaire-based factors and the 313-variant PRS were previously obtained from a literature review as described by ref. ^8^ (supplementary Table 1). The relative risk estimate of the four-level density variable was obtained through a literature search and was integrated assuming a multiplicative joint association with the other factors^57^. For women younger than 50, the estimate was obtained from ref. ^19^, which was adjusted for age. For women over 50 years, the estimate was obtained from ref. ^58^, and was adjusted for age, BMI, menopausal status, surgical menopause, family history, age at childbirth, HRT use, prior breast procedures, race, Hispanic ethnicity, and previous mammographic outcome.

An individual-level reference dataset of risk factors, representative of the underlying country’s population, was used to estimate the joint distribution of risk factors and absolute risk projections in the respective population. For the US population, the majority of the risk factors were derived from the National Health and Nutrition Examination Survey (NHANES) from 2008, 2010, and 2012 as previously described^8^. The PRS was simulated assuming an independent relationship with the questionnaire-based risk factors, conditional on family history. Density was incorporated into the existing reference dataset by estimating the joint distribution of classical risk factors and density using proportional odds regression methods for ordinal data. Analyses were performed in the Breast Cancer Surveillance Consortium (BCSC) dataset, and data were collected from six participating registries: KP Washington, New Hampshire, North Carolina, San Francisco, Vermont and Chicago. Our dataset included 815,968 US women of European-ancestry aged 30–70 with screening mammograms and diagnostic mammograms, collected from 2005 through 2017. For each woman, the first record and corresponding covariates collected at that visit were used, and only records with complete covariate information were included in the analysis. The majority of the risk factors in the BCSC dataset were obtained through a questionnaire filled out by patients (available from www.bcsc-research.org). The variable on benign breast disease was derived from pathology reports and included the diagnosis with the highest severity. Finally, 144,037 women were included with full risk-factor information, from which we simulated the density variable for the reference dataset.

For the Swedish population, the reference dataset was generated using data on subjects with no diagnosis of breast cancer and with no missing information for the risk-factor variables from the prospective cohort KARMA. The sources for building the reference datasets for each country may be found in Table S4.

Lastly, the marginal age-specific disease incidence rates are provided by the United States’ Surveillance Epidemiology and End Results (SEER) cancer registry, 2008–2012. Competing mortality rates due to causes other than breast cancer was obtained from the CDC WONDER database 2008–2012.

### Model validation

We evaluated models predicting 5-year absolute breast cancer risk prospectively for calibration and discriminatory accuracy. We considered models with only questionnaire-based risk factors, questionnaire-based risk factors and density, questionnaire-based risk factors and PRS, a model with density and PRS, and lastly, a fully integrated model with questionnaire-based risk factors, PRS and density.

Calibration refers to the model’s ability to accurately predict the absolute risk of breast cancer. We evaluate calibration by estimating the ratio of expected-to-observed (E/O) number of cases, the calibration slope and intercept. To assess calibration, the individual subjects were stratified in low to high-risk categories based on deciles of both predicted 5-year absolute risk and their relative risk score. For each category, the average predicted absolute risk and relative risk scores were computed. The E/O ratio was calculated overall (and within each category), first using the predicted absolute risk. The expected-to-observed ratio is defined as the ratio of the average expected absolute risk and the proportion of observed cases, overall and by category. We assessed model calibration using the Hosmer-Lemeshow (HL) goodness-of-fit test, where the HL statistic approximately follows a *χ*^2^ distribution. We also estimated a calibration slope and intercept from a linear regression of the observed and expected proportion of cases within risk deciles.

The discriminatory accuracy of the model is assessed using the area under the receiver operating characteristic curve (AUC) based on the 5-year absolute risk and relative risk scores. AUC is defined as the probability that the risk for a randomly selected case is higher than the risk for a randomly selected control. An AUC of 50% corresponds to a model with no discriminatory power, while 100% corresponds to perfect discrimination. The AUC was estimated empirically as the proportion of case-control pairs in which the risk of the case is higher than that of the control. The 95% Wald-based confidence intervals for AUC using the asymptotic variance formula were computed^59^.

### Adjustment for nested sampling

The fully integrated model incorporated a 313-SNP PRS and BI-RADS breast density. In all validation cohorts, BI-RADS density and the genotype data on SNPs used to calculate the PRS was available for a nested case-control study. To reflect the underlying full cohort, all calculations are adjusted using sampling weights using the inverse-probability weighted (IPW) approach. Weights are defined by the probability of selection of subjects to the nested case-control sample from the full cohort. The probability of selection is estimated by fitting a logistic regression model with age of study entry, follow-up time, the matching factors, and interactions of case/control status with age of study entry and follow-up time to the entire underlying cohort, where the outcome is defined as whether the individual is included or not. All standard errors and 95% Wald-based confidence intervals were calculated using influence function-based variance estimators that account for sampling weights.

### Risk reclassification

Improvements in risk stratification resulting from the incorporation of density to a model with questionnaire-based risk factors and PRS were assessed among women of European-ancestry aged 50–70 years in the populations of the US and Sweden by calculating the number of women and future cases identified to be at high-risk based on pre-specified 5-year absolute risk thresholds. We used two high-risk thresholds: 3%, which corresponds to the United States Preventive Services Task Force (USPSTF) recommendation for risk-reducing interventions^60^, as well as 6%, which corresponds to the breast cancer risk of a BRCA mutation carrier and is used as a cutoff for very high risk by the WISDOM Trial^24,25^. We also calculated the number of women and future cases reclassified at the high-risk thresholds to further quantify the improvements in risk stratification after incorporating density into a model with questionnaire-based risk factors and PRS.

All analyses were performed using R version 3.6.2 (www.r-project.org).

### Ethics declaration

All contributing studies received approval from their respective local institutional review boards. Study participants provided informed consent, where applicable, and participated under protocols approved by ethics committees. The corresponding ethics approvals are as follows: MMHS—approved at Mayo Clinic under 08-000847; KARMA— approved by the Swedish Ethical Review Authority under 2010/958-31/1; NHS—approved at Mass General Brigham under 1999P002112.

## Supplementary information

Supplementary Information

## Data Availability

The datasets used in the current analysis will not be made publicly available due to restraints imposed by the ethics committees of individual studies; requests for individual-level data for all the participants in the full cohort of any study can be made to the individual studies.

## References

[1] Chatterjee, N., Shi, J. & García-Closas, M. Developing and evaluating polygenic risk prediction models for stratified disease prevention. *Nat. Rev. Genet*. **17**, 392–406 (2016). 10.1038/nrg.2016.27 PMC6021129 27140283

[2] Garcia-Closas, M. & Chatterjee, N. Assessment of breast cancer risk: which tools to use? *Lancet Oncol*. **20**, 463–464 (2019). 10.1016/S1470-2045(19)30071-3 PMC8211385 30799258

[3] Louro, J. et al. A systematic review and quality assessment of individualised breast cancer risk prediction models. *Br. J. Cancer*. **121**, 76-85 (2019). 10.1038/s41416-019-0476-8 PMC6738106 31114019

[4] Cintolo-Gonzalez, J. A. et al. Breast cancer risk models: a comprehensive overview of existing models, validation, and clinical applications. *Breast Cancer Res. Treat.* **164**, 263–284 (2017). 10.1007/s10549-017-4247-z 28444533

[5] Mavaddat, N. et al. Polygenic risk scores for prediction of breast cancer and breast cancer subtypes *Am. J. Hum. Genet.* **104**, 21–34 (2019). 10.1016/j.ajhg.2018.11.002 PMC6323553 30554720

[6] Pal Choudhury, P. et al. iCARE: an R package to build, validate and apply absolute risk models *PLoS ONE* **15**, e0228198 (2020). 10.1371/journal.pone.0228198 PMC7001949 32023287

[7] Hurson, A. N. et al. Prospective evaluation of a breast-cancer risk model integrating classical risk factors and polygenic risk in 15 cohorts from six countries. *Int. J. Epidemiol.* **50**, 1897–1911 (2021). 10.1093/ije/dyab036 PMC8743128 34999890

[8] Choudhury, P. P. et al. Comparative validation of breast cancer risk prediction models and projections for future risk stratification. *J. Natl Cancer Inst.* **112**, 278–285 (2020). 10.1093/jnci/djz113 PMC7073933 31165158

[9] Wolfe, J. Breast patterns as an index of risk for developing breast cancer. *Am. J. Roentgenol.* **126**, 1130–1137 (1976). 179369 10.2214/ajr.126.6.1130

[10] Boyd, N. F. et al. Mammographic density and the risk and detection of breast cancer. *N. Engl. J. Med.* **356**, 227–236 (2007). 10.1056/NEJMoa062790 17229950

[11] *Breast Imaging Reporting and Data System (BI-RADS)*. 5th edn (American College of Radiology, 2013).

[12] McCormack, V. A. & Dos Santos Silva, I. Breast density and parenchymal patterns as markers of breast cancer risk: a meta-analysis. *Cancer Epidemiol. Biomarkers Prev.* **15**, 1159–1169 (2006). 10.1158/1055-9965.EPI-06-0034 16775176

[13] Bertrand, K. A. et al. Mammographic density and risk of breast cancer by age and tumor characteristics. *Breast Cancer Res.* **15**, R104 (2013). 10.1186/bcr3570 PMC3978749 24188089

[14] Vachon, C. M. et al. Mammographic density, breast cancer risk and risk prediction. *Breast Cancer Res.* **9**, 217 (2007). 18190724 10.1186/bcr1829 PMC2246184

[15] Boyd, N. F. et al. Mammographic density: a heritable risk factor for breast cancer. *Methods Mol. Biol.* **472**, 343–360 (2009). 10.1007/978-1-60327-492-0_15 19107441

[16] Tice, J. A. et al. Validation of the breast cancer surveillance consortium model of breast cancer risk. *Breast Cancer Res. Treat.* **175**, 519–523 (2019). 10.1007/s10549-019-05167-2 PMC7138025 30796654

[17] Tice, J. A. et al. Using clinical factors and mammographic breast density to estimate breast cancer risk: development and validation of a new predictive model. *Ann. Intern. Med.* **148**, 337–347 (2008). 10.7326/0003-4819-148-5-200803040-00004 PMC2674327 18316752

[18] Tice, J. A., Cummings, S. R., Ziv, E. & Kerlikowske, K. Mammographic breast density and the Gail model for breast cancer risk prediction in a screening population. *Breast Cancer Res. Treat.* **94**, 115–122 (2005). 10.1007/s10549-005-5152-4 16261410

[19] Tice, J. A. et al. Breast density and benign breast disease: risk assessment to identify women at high risk of breast cancer. *J. Clin. Oncol.* **33**, 3137–3143 (2015). 10.1200/JCO.2015.60.8869 PMC4582144 26282663

[20] Vachon, C. M. et al. The contributions of breast density and common genetic variation to breast cancer risk. *J. Natl Cancer Inst.* **107**, dju397 (2015). 10.1093/jnci/dju397 PMC4598340 25745020

[21] Chen, J. et al. Projecting absolute invasive breast cancer risk in White women with a model that includes mammographic density. *J. Natl Cancer Inst.* **98**, 1215–1226 (2006). 10.1093/jnci/djj332 16954474

[22] Brentnall, A. R., Cuzick, J., Buist, D. S. M. & Bowles, E. J. A. Long-term accuracy of breast cancer risk assessment combining classic risk factors and breast density. *JAMA Oncol.* **4**, e180174 (2018). 10.1001/jamaoncol.2018.0174 PMC6143016 29621362

[23] Warwick, J. et al. Mammographic breast density refines Tyrer-Cuzick estimates of breast cancer risk in high-risk women: findings from the placebo arm of the International Breast Cancer Intervention Study I. *Breast Cancer Res.* **16**, 451 (2014). 10.1186/s13058-014-0451-5 PMC4303130 25292294

[24] Shieh, Y. et al. Breast cancer screening in the precision medicine era: risk-based screening in a population-based trial. *J. Natl Cancer Inst.*10.1093/jnci/djw290 (2017). 10.1093/jnci/djw290 28130475

[25] Eklund, M. et al. The WISDOM personalized breast cancer screening trial: simulation study to assess potential bias and analytic approaches. *JNCI Cancer Spectr.*10.1093/jncics/pky067 (2019). 10.1093/jncics/pky067 PMC6649825 31360882

[26] MyPeBS personalized breast screening. https://www.mypebs.eu/ (2020).

[27] Esserman, L. J. The WISDOM Study: breaking the deadlock in the breast cancer screening debate. *npj Breast Cancer* **3**, 34 (2017). 10.1038/s41523-017-0035-5 PMC5597574 28944288

[28] Hart, V. et al. The effect of change in body mass index on volumetric measures of mammographic density. *Cancer Epidemiol. Biomark. Prev.* **24**, 1724–1730 (2015). 10.1158/1055-9965.EPI-15-0330 PMC4633314 26315554

[29] McHugh, M. L. Interrater reliability: the kappa statistic. *Biochem. Med.* **22**, 276–282 (2012). PMC3900052 23092060

[30] Berg, W. A., Campassi, C., Langenberg, P., Sexton, M. J. Breast Imaging Reporting and Data System: inter- and intraobserver variability in feature analysis and final assessment. *AJR Am J Roentgenol.* **174**, 1769–77 (2000). 10.2214/ajr.174.6.1741769 10845521

[31] Gard, C. C., Aiello Bowles, E. J., Miglioretti, D. L., Taplin, S. H. & Rutter, C. M. Misclassification of breast imaging reporting and data system (BI-RADS) mammographic density and implications for breast density reporting legislation. *Breast J.* **21**, 481–489 (2015). 26133090 10.1111/tbj.12443 PMC4558212

[32] Kerlikowske, K. et al. Variability and accuracy in mammographic interpretation using the American College of Radiology Breast Imaging Reporting and Data System. *J. Natl Cancer Inst.* **90**, 1801–1809 (1998). 10.1093/jnci/90.23.1801 9839520

[33] Spayne, M. C. et al. Reproducibility of BI-RADS breast density measures among community radiologists: a prospective cohort study. *Breast J.* **18**, 326–326 (2012). 22607064 10.1111/j.1524-4741.2012.01250.x PMC3660069

[34] Kerlikowske, K. et al. Automated and clinical breast imaging reporting and data system density measures predict risk for screen-detected and interval cancers: a case-control study. *Ann. Intern. Med.* **168**, 757–765 (2018). 10.7326/M17-3008 PMC6447426 29710124

[35] Brentnall, A. R. et al. A case-control study to add volumetric or clinical mammographic density into the Tyrer-Cuzick breast cancer risk model. *J. Breast Imaging* **1**, 99–106 (2019). 10.1093/jbi/wbz006 PMC6690422 31423486

[36] Institute, N. C. Connect for cancer prevention study. (2025) https://www.cancer.gov/connect-prevention-study/.

[37] Yang, X. et al. Prospective validation of the BOADICEA multifactorial breast cancer risk prediction model in a large prospective cohort study. *J. Med. Genet.* **59**, 1196–1205 (2022). 10.1136/jmg-2022-108806 PMC9691822 36162852

[38] Van Veen, E. M. et al. Use of single-nucleotide polymorphisms and mammographic density plus classic risk factors for breast cancer risk prediction. *JAMA Oncol.* **4**, 476–482 (2018). 29346471 10.1001/jamaoncol.2017.4881 PMC5885189

[39] Brentnall, A. R., et al. A case-control evaluation of 143 single nucleotide polymorphisms for breast cancer risk stratification with classical factors and mammographic density. *Int. J. Cancer* **146**, 2122–2129 (2020). 31251818 10.1002/ijc.32541 PMC7065068

[40] Zhang, X. et al. Addition of a polygenic risk score, mammographic density, and endogenous hormones to existing breast cancer risk prediction models: a nested case–control study. *PLoS Med.* **15**, e1002644 (2018). 10.1371/journal.pmed.1002644 PMC6122802 30180161

[41] Brentnall, A. R. et al. Mammographic density adds accuracy to both the Tyrer-Cuzick and Gail breast cancer risk models in a prospective UK screening cohort. *Breast Cancer Res.* **17**, 147 (2015). 10.1186/s13058-015-0653-5 PMC4665886 26627479

[42] Evans, D. G. R. et al. Breast cancer pathology and stage are better predicted by risk stratification models that include mammographic density and common genetic variants. *Breast Cancer Res. Treat.* **176**, 141–148 (2019). 10.1007/s10549-019-05210-2 PMC6548748 30941651

[43] Lee, A. et al. BOADICEA: a comprehensive breast cancer risk prediction model incorporating genetic and nongenetic risk factors. *Genet. Med.* **21**, 1708–1718 (2019). 10.1038/s41436-018-0406-9 PMC6687499 30643217

[44] Destounis, S., Santacroce, A. & Arieno, A. *Am. J. Roentgenol.* **214**, 296–305 (2020). 10.2214/AJR.19.21994 31743049

[45] Haji Maghsoudi, O. et al. Deep-LIBRA: an artificial-intelligence method for robust quantification of breast density with independent validation in breast cancer risk assessment *Med. Image Anal.* **73**, 102138 (2021). 10.1016/j.media.2021.102138 PMC8453099 34274690

[46] Gastounioti, A., Desai, S., Ahluwalia, V. S., Conant, E. F. & Kontos, D. Artificial intelligence in mammographic phenotyping of breast cancer risk: a narrative review. *Breast Cancer Res.*10.1186/s13058-022-01509-z (2022). 10.1186/s13058-022-01509-z PMC8859891 35184757

[47] Hudson, S. M., Wilkinson, L. S., De Stavola, B. L. & dos-Santos-Silva, I. Left-right breast asymmetry and risk of screen-detected and interval cancers in a large population-based screening population. *Br. J. Radiol.*10.1259/BJR.20200154/7448900 (2020). 10.1259/bjr.20200154 PMC7446006 32525693

[48] Vachon, C. M. et al. Impact of artificial intelligence system and volumetric density on risk prediction of interval, screen-detected, and advanced breast cancer. *J. Clin. Oncol.* **41**, 3172–3183 (2023). 37104728 10.1200/JCO.22.01153 PMC10256336

[49] Barnett, G. C. et al. Risk factors for the incidence of breast cancer: do they affect survival from the disease?. *J. Clin. Oncol.* **26**, 3310–3316 (2008). 18612147 10.1200/JCO.2006.10.3168

[50] Olson, J. E. et al. The influence of mammogram acquisition on the mammographic density and breast cancer association in the mayo mammography health study cohort. *Breast Cancer Research* **14**, (2012). 10.1186/bcr3357 PMC3701143 23152984

[51] Boyd, N. F. et al. Quantitative classification of mammographic densities and breast cancer risk: results from the Canadian National Breast Screening Study. *J. Natl Cancer Inst.* **87**, 670–675 (1995). 10.1093/jnci/87.9.670 7752271

[52] Eriksson, M., Li, J., Leifland, K., Czene, K. & Hall, P. A comprehensive tool for measuring mammographic density changes over time. *Breast Cancer Res. Treat.* **169**, 371–379 (2018). 29392583 10.1007/s10549-018-4690-5 PMC5945741

[53] Spak, D. A., Plaxco, J. S., Santiago, L., Dryden, M. J. & Dogan, B. E. BI-RADS^®^ fifth edition: a summary of changes. *Diagn. Interv. Imaging* **98**, 179–190 (2017). 10.1016/j.diii.2017.01.001 28131457

[54] Astley, S. M. et al. A comparison of five methods of measuring mammographic density: a case-control study. *Breast Cancer Res.* **20**, 10 (2018). 10.1186/s13058-018-0932-z PMC5799922 29402289

[55] Fowler, E. E., Sellers, T. A., Lu, B. & Heine, J. J. Breast imaging reporting and data system (BI-RADS) breast composition descriptors: automated measurement development for full field digital mammography. *Med. Phys.* **40**, 113502 (2013). 10.1118/1.4824319 PMC3820635 24320473

[56] Eriksson, M. et al. A clinical model for identifying the short-term risk of breast cancer. *Breast Cancer Res.*10.1186/s13058-017-0820-y (2017). 10.1186/s13058-017-0820-y PMC5348894 28288659

[57] Vachon, C. M. et al. Joint association of mammographic density adjusted for age and body mass index and polygenic risk score with breast cancer risk. *Breast Cancer Res.* **21**, 68 (2019). 31118087 10.1186/s13058-019-1138-8 PMC6532188

[58] Barlow, W. E. et al. Prospective Breast Cancer Risk Prediction Model for Women Undergoing Screening Mammography. *J. Natl. Cancer Inst.* **98**, 1204–1663 (2006). 16954473 10.1093/jnci/djj331

[59] DeLong, E. R., DeLong, D. M. & Clarke-Pearson, D. L. Comparing the areas under two or more correlated receiver operating characteristic curves: a nonparametric approach. *Biometrics* **44**, 837–845 (1988). 3203132

[60] Owens, D. K. et al. Medication use to reduce risk of breast cancer: US Preventive Services Task Force recommendation statement. *JAMA* 322, 857–867 (2019). 10.1001/jama.2019.11885 31479144

