# Supplementary material for: Evaluating mammographic density′s contribution to improve a breast cancer risk model with questionnaire-based and polygenic factors
Source: NPJ Breast Cancer. 2025 Oct 1;11:106. doi: 10.1038/s41523-025-00813-z (PMC12488985; doi:10.1038/s41523-025-00813-z)
Supplement: Supplementary file 1 — Supplementary Information [file 41523_2025_813_MOESM1_ESM.pdf]

Supplementary Materials

Investigating the added value of incorporating mammographic density to an integrated breast cancer risk model with questionnaire-based risk factors and polygenic risk score

Table of Contents **Page**

**Table S1**	Characteristics of prospective cohort studies used for model validation	**2**
**Table S2**	Breast cancer risk factor distribution by cohort	**3**
**Table S3**	Sources of information for model inputs for each country	**5**
**Table S4**	Breast cancer risk factor distribution in each reference population dataset	**6**
**Table S5**	Model calibration measured by expected-to-observed ratios of various iCARE-Lit models in women younger than 50 and 50 years and older.	**8**
**Table S6A**	Risk discrimination measured by the model area under the curve of various iCARE-Lit models in women younger than 50	**9**
**Table S6B**	Risk discrimination measured by the model area under the curve of various iCARE-Lit models in women 50 years and older	**10**
**Table S7**	Proportion of women reclassified at the high-risk thresholds based on risk estimated from the integrated model with questionnaire-based risk factors, 313-SNP PRS and BI-RADS, as opposed to the model with questionnaire-based risk factors and 313-SNP PRS only	**11**
**Table S8**	Reclassification at high-risk thresholds after incorporating BI-RADS density to questionnaire-based risk factors and 313-SNP PRS	**12**
**Table S9**	Overview of validation studies of breast cancer risk models including polygenic-risk-score and mammographic breast density	**13**
**Figure S1A**	Absolute risk and relative risk calibration in KARMA for women younger than 50 years	**14**
**Figure S1B**	Absolute risk and relative risk calibration in NHS II for women younger than 50 years	**15**
**Figure S1C**	Absolute risk and relative risk calibration in KARMA for women 50 years and older	**16**
**Figure S1D**	Absolute risk and relative risk calibration in NHS I for women 50 years and older	**17**
**Figure S1E**	Absolute risk and relative risk calibration in MMHS for women 50 years and older	**18**

Table S1. Characteristics of prospective cohort studies used for model validation

Study	Country	Full cohort [b]					Nested cohort [b, c]	
		Year(s) at DNA collection	Age at DNA collection [a]	Years of follow-up [a]	Cases	Total	Cases	Controls
NHS II	US	1996	43 (33-49)	13 (7-15)	390	57 695	142	941
KARMA	Sweden	2011-2013	45 (27-49)	5 (3-7)	179	24 018	138	4096
			60 (50-72)	5 (3-7)	683	42 280	557	10616
MMHS	US	2003-2006	61 (50-75)	9 (0-10)	269	11 996	242	1040
NHS I	US	1989	64 (50-75)	14 (9-21)	1245	58 163	389	2411

[a] Mean (range)
[b] Defined as women of European ancestry, with no history of breast cancer, who provided a sample of DNA
[c] Includes subjects with genotype data and BI-RADS data

Table S2. Breast cancer risk factor distribution by cohort

Breast cancer risk factors	MMHS N=11,996	NHS I N=65,926	NHS II N=58,940	KARMA N=65,436
**Age at baseline, years**				
Median (IQR)	60 (54, 66)	65 (59,71)	44 (41,47)	
**Age at menarche, N (%)**				
≤11	2,114 (18)	13,156 (22)	8328 (22)	13,049 (20)
12 – 13	6,422 (57)	33,169 (57)	21 103 (58)	29,774 (46)
14 – 15	2,302 (20)	9,812 (16)	5591 (15)	19,692 (31)
≥16	366 (3.3)	2,026 (3.5)	1301 (3.6)	2,921 (4.5)
Missing	792	0	0	0
**Parity, N (%)**				
Nulliparous	1,428 (12)	3,181 (5.6)	6533 (23)	7,762 (13)
1 birth	998 (8.4)	3,740 (6.5)	4593 (17)	8,916 (15)
2 births	3,678 (31)	15,290 (27)	14 061 (51)	29,305 (48)
3 + births	5,742 (49)	35,098 (61)	2681 (9.6)	15,163 (25)
Missing	150	854	8455	4290
**Age at first birth, years (among parous women), N (%)**				
<20	2,221 (21)	411 (0.8)	1041 (5.8)	3,108 (5.8)
20 – 24	5,208 (50)	27,919 (52)	3568 (20)	14,915 (30)
25 – 29	2,231 (21)	20,526 (38)	7298 (41)	18,847 (35)
≥30	748 (7.2)	52,58 (9.7)	6054 (34)	16,625 (31)
Missing	10	14	3374	22
**OC use (among subjects age <50), N (%)**				
Never	---	---	4729 (13)	---
Former	---	---	29 472 (82)	---
Current	---	---	1834 (5.1)	---
Missing	---	---	288	---
**OC use (among subjects age ≥50), N (%)**				
Never	3,075 (26)	29,752 (52)	---	13689 (21)
Ever	8,843 (72)	27,294 (48)	---	51747 (79)
Missing	78	1,117	---	0
**HRT use (among subjects age ≥50), N (%)**				
Never	4,056 (38)	14,650 (29)	---	46106 (81)
Former	3,829 (36)	15,624 (30)	---	8921 (16)
Current	2,803 (26)	21,105 (41)	---	1741 (3)
Missing	1,308	6,784	---	8668
**Type of HRT use (among current users age ≥50), N (%)**				
Current E-type	518 (27)	7,373 (40)	---	815 (47)
Current C-type	1,418 (73)	11,232 (60)	---	926 (53)
Missing	867	39,558	---	8668
**Age at menopause, years (among subjects age ≥50), N (%)**				
< 40	702 (10)	2,234 (4.2)	---	717 (4)
40 – 44	847 (13)	3,748 (7.0)	---	1460 (9)
45 – 49	1,838 (27)	13,449 (25)	---	4106 (25)
50 – 54	2,597 (38)	30,486 (57)	---	7684 (46)
≥55	771 (11)	3,914 (7.3)	---	2624 (16)
Missing	5,241	4,332	---	48845

Breast cancer risk factors	MMHS n (%) N=11,996	NHS I n (%) N=58,163	NHS II N=36,323	KARMA N=65,436
**Height, cm**				
Median (IQR)	163 (IQR: (159, 168))	163 (160, 168)	165 (160, 170)	166 (160, 172)
**Body mass index (kg/m²), N (%)**				
<25	3,843 (32)	26,436 (48)	19,510 (55)	38,113 (58)
≥25 - <30	4,009 (34)	18,001 (33)	9,107 (26)	19,402 (30)
≥30	4,131 (35)	10,383 (19)	7,138 (20)	7,921 (12)
Missing	13	3,343	568	0
**Alcohol (g/day), N (%)**				
None	3,975 (33)	22,346 (40)	13,321 (37)	11,708 (19)
<5	6,283 (53)	19,531 (35)	15,004 (42)	15,726 (26)
5 – 14	1,160 (9.7)	9,111 (16)	5,753 (16)	24,673 (41)
15 – 24	353 (3.0)	3,188 (5.7)	1,087 (3.0)	5,900 (10)
25 – 34	126 (1.1)	939 (1.7)	427 (1.2)	1,209 (2)
35 – 44	19 (0.2)	1 (0.0)	196 (0.5)	1,195 (2)
≥45	2 (0.0)	548 (0.1)	137 (0.4)	218 (0.4)
Missing	78	2,499	398	4,807
**History of BBD, N (%)**				
No	8,931 (74)	37,310 (64)	10,837 (64)	46399 (78)
Yes	3,065 (26)	20,852 (36)	6,109 (36)	13296 (22)
Missing	0	1	19,377	5741
**Family history of breast cancer in first degree relatives, N (%)**				
No	9,666 (81)	50,191 (86)	33 064 (91)	57975 (89)
Yes	2,330 (19)	7,972 (14)	3259 (9)	7461 (11)
Missing	0	0	0	0
**BI-RADS density [a], N (%)**				
a	2,984 (25)	491 (20)	50 (5)	8,191 (13)
b	5,120 (43)	873 (36)	169 (18)	24,493 (37)
c	3,333 (28)	783 (32)	397 (42)	25,915 (40)
d	546 (5)	264 (11)	325 (35)	6,837 (10)
Missing	0	0	0	0

Abbreviations: BBD = benign breast disease, C-type = estrogen and progestogen combined, E-type = estrogen-only, HRT = hormone replacement therapy, IQR = interquartile range, OC = oral contraceptive.
[a] For NHSI and NHSII, there is no full-cohort information on BI-RADS density. Controls-only data from the nested case-control study is reported.

Table S3. Sources of information for model inputs for each country

Country	Breast Cancer Incidence	Competing Mortality	Reference Risk Factor Distribution
Sweden	The National Board of Health and Welfare, 2016	The National Board of Health and Welfare, 2016	Controls of KARMA cohort
United States	US National Cancer Institute-Surveillance, Epidemiology, and End Results Program, 2008-2012	Center for Disease Control WONDER database, 2008-2012	Questionnaire-based risk factors, as previously described[1]:<br>- National Health and Nutrition Examination Survey, 2008, 2010, 2012<br>- Women's Health Initiative, 2010<br>- National Health Interview Survey, 2010<br>- PLCO cohort<br><br>BI-RADS:<br>- Breast Cancer Surveillance Consortium, 2005-2017

Table S4. Breast cancer risk factor distribution in each reference population dataset

Risk Factors	United States	Sweden
**Age at first birth, years (among parous women)**		
<20	26.8%	4.7%
20 – 24	38.9%	22.7%
25 – 29	21.7%	28.7%
≥30	12.6%	25.3%
**OC use**		
Never	19.1	20.9%
Ever	80.9	79.1%
**Height, cm**		
Mean	162.3 (SD: 6.4)	166.6 (SD: 6.0)
**Alcohol, g/day**		
None	45%	17.9%
<5	39.7%	24.0%
5 – 14	12.1%	37.7%
15 – 24	2.3%	9.0%
25 – 34	0.6%	1.8%
35 – 44	0.2%	1.8%
≥45	0.2%	0.3%
**History of BBD**		
No	83.3%	74.3%
Yes	16.8%	25.7%
**Family history of breast cancer in first degree relatives**		
No	86.4%	88.6%
Yes	13.6%	11.4%
**Age at menarche, years**		
≤11	33.3%	19.9%
12 – 13	43.8%	45.5%
14 – 15	20.1%	30.1%
≥16	2.6%	4.5%
**Age at menopause, years**		
< 40	21.3%	1.6%
40 – 44	13.3%	2.9%
45 – 49	22.7%	10.2%
50 – 54	30.3%	78.5%
≥55	12.4%	6.8%
**Parity**		
Nulliparous	17.6	11.9%
1 birth	15.1	13.6%
2 births	33.7	44.8%
3 + births	33.6	23.2%

Risk Factors	United States	Sweden
**Body mass index, kg/m^2**		
<25<br>≥25 - <30<br>≥30	35.1%<br>31.9%<br>33.1%	58.3%<br>29.7%<br>12.1%
**BMI and use of HRT**		
BMI <25: Never HRT use	20.4%	32.2%
BMI 25-29: Never HRT use	18.4%	20.4%
BMI ≥30: Never HRT use	19.5%	8.5%
BMI <25: Former HRT use	10.5%	17.9%
BMI 25-29: Former HRT use	10.2%	11.5%
BMI ≥30: Former HRT use	11.1%	4.0%
BMI <25: Current use of C-type HRT	1.8%	3.0%
BMI 25-29: Current use of C-type HRT	1.4%	2.0%
BMI ≥30: Current use of C-type HRT	1.1%	0.6%
BMI <25: Current use of E-type HRT	2.3%	2.2%
BMI 25-29: Current use of E-type HRT	1.8%	1.5%
BMI ≥30: Current use of E-type HRT	1.3%	0.5%
**BI-RADS**		
a<br>b<br>c<br>d	11.9%<br>54.7%<br>28.3%<br>5.2%	12.5%<br>37.4%<br>39.6%<br>10.4%

Table S5. Model calibration measured by expected-to-observed ratios of various iCARE-Lit models in women younger than 50 and 50 years and older.

Population and model			Number of controls / cases	Overall E/O (95 %CI)
Women younger than 50 years	KARMA	QRF only	4,096/ 138	1.34 (1.13-1.58)
		PRS only		1.10 (0.932-1.31)
		QRF + MBD		1.10 (0.933-1.31)
		PRS + MBD		1.10 (0.93-1.30)
		QRF+ PRS		1.12 (0.95-1.33)
		QRF + PRS + MBD		1.12 (0.95-1.33)
	NHS II	QRF only	941/ 142	0.80 (0.64-1.00)
		PRS only		0.68 (0.54-0.84)
		QRF + MBD		0.80 (0.64-1.00)
		PRS + MBD		0.68 (0.55-0.85)
		QRF+ PRS		0.83 (0.67-1.04)
		QRF + PRS + MBD		0.85 (0.68-1.06)
Women 50 years and older	KARMA	QRF only	10,616/ 557	0.89 (0.82-0.96)
		PRS only		0.83 (0.76-0.90)
		QRF + MBD		0.83 (0.76-0.90)
		PRS + MBD		0.87 (0.80-0.95)
		QRF+ PRS		0.86 (0.79-0.93)
		QRF + PRS + MBD		0.87 (0.80-0.94)
	NHS I	QRF only	2,411/ 389	0.82 (0.74-0.93)
		PRS only		0.78 (0.70-0.88)
		QRF + MBD		0.82 (0.73-0.92)
		PRS + MBD		0.77 (0.69-0.87)
		QRF+ PRS		0.86 (0.77-0.97)
		QRF + PRS + MBD		0.87 (0.78-0.98)
	MMHS	QRF only	1,040 / 242	0.95 (0.77-1.18)
		PRS only		0.78 (0.63-0.97)
		QRF + MBD		0.89 (0.77-1.02)
		PRS + MBD		0.77 (0.67-0.89)
		QRF+ PRS		0.89 (0.77-1.10)
		QRF + PRS + MBD		0.89 (0.77-1.02)

The iCARE-Lit models risk-factor combinations are: (i) questionnaire-based risk factors and BI-RADS breast density, (ii) BI-RADS breast density with the 313-SNP PRS, (iii) the 313-SNP PRS and questionnaire-based risk factors, and (iv) the fully integrated model incorporating questionnaire-based risk factors, the 313-SNP PRS and BI-RADS breast density. Abbreviations: AUC = area under the curve; CI = confidence interval; E/O = ratio of expected to observed absolute risk; MBD = Mammographic Breast Density; MMHS = Mayo Mammography Health Study; PRS = Polygenic Risk Score; QRF = questionnaire-based risk factors

Table S6A. Risk discrimination measured by the model area under the curve of various iCARE-Lit models in women younger than 50 and risk discrimination measured by the model area under the curve based on a meta-analysis across both studies.

Population and model		Number of controls / cases	AUC (95% CI)
KARMA	QRF only	4,096/ 138	58.8 (54.2-63.3)
	PRS only		62.7 (57.9-67.6)
	QRF + MBD		63.2 (58.8-67.7)
	PRS + MBD		65.3 (60.6-70.0)
	QRF+ PRS		64.2 (59.6-68.9)
	QRF + PRS + MBD		66.4 (61.9-70.9)
NHS II	QRF only	941/ 142	62.2 (55.7-68.7)
	PRS only		64.9 (59.2-70.6)
	QRF + MBD		63.4 (57.5-69.4)
	PRS + MBD		66.4 (60.7-72.0)
	QRF+ PRS		67.5 (61.4-73.7)
	QRF + PRS + MBD		68.2 (62.3-74.0)
Overall	QRF only	5,037/ 280	59.9 (56.2-63.6)
	PRS only		63.6 (59.9, 67.3)
	QRF + MBD		63.3 (59.7- 66.8)
	PRS + MBD		65.7 (62.1, 69.4)
	QRF+ PRS		65.4 (61.7, 69.1)
	QRF + PRS + MBD		67.1 (63.5, 70.6)

The iCARE-Lit models risk-factor combinations are: (i) questionnaire-based risk factors and BI-RADS breast density, (ii) BI-RADS breast density with the 313-SNP PRS, (iii) the 313-SNP PRS and questionnaire-based risk factors, and (iv) the fully integrated model incorporating questionnaire-based risk factors, the 313-SNP PRS and BI-RADS breast density. Abbreviations: AUC = area under the curve; CI = confidence interval; E/O = ratio of expected to observed absolute risk; MBD = Mammographic Breast Density; MMHS = Mayo Mammography Health Study; PRS = Polygenic Risk Score; QRF = questionnaire-based risk factors

Table S6B. Risk discrimination measured by the model area under the curve of various iCARE-Lit models in women 50 years and older and risk discrimination measured by the model area under the curve based on a meta-analysis across all studies.

Population and model		Number of controls / cases	AUC (95% CI)
KARMA	QRF only	10,616/ 557	61.0 (58.7-63.4)
	PRS only		64.0 (61.7-66.3)
	QRF + MBD		61.8 (59.4-64.1)
	PRS + MBD		64.7 (62.5-67.0)
	QRF+ PRS		65.5 (63.2-67.8)
	QRF + PRS + MBD		66.1 (63.9-68.4)
NHS I	QRF only	2,411/ 389	58.8 (55.5-62.0)
	PRS only		62.1 (58.8-65.4)
	QRF + MBD		62.4 (59.1-65.6)
	PRS + MBD		65.1 (61.7-68.4)
	QRF+ PRS		65.3 (62.1-68.5)
	QRF + PRS + MBD		67.2 (63.9-70.5)
MMHS	QRF only	1,040 / 242	56.5 (52.0-61.1)
	PRS only		65.6 (61.4-69.8)
	QRF + MBD		57.5 (52.9-62.1)
	PRS + MBD		63.4 (59.0-67.9)
	QRF+ PRS		65.9 (61.7-70.0)
	QRF + PRS + MBD		63.9 (59.5-68.3)
Overall	QRF only	14,067/ 1,188	59.7 (57.9-61.4)
	PRS only		63.8 (62.0-65.5)
	QRF + MBD		61.3 (59.6-63.1)
	PRS + MBD		64.6 (62.9, 66.3)
	QRF+ PRS		65.5 (63.8-67.2)
	QRF + PRS + MBD		66.1 (64.4-67.8)

The iCARE-Lit models risk-factor combinations are: (i) questionnaire-based risk factors and BI-RADS breast density, (ii) BI-RADS breast density with the 313-SNP PRS, (iii) the 313-SNP PRS and questionnaire-based risk factors, and (iv) the fully integrated model incorporating questionnaire-based risk factors, the 313-SNP PRS and BI-RADS breast density. Abbreviations: AUC = area under the curve; CI = confidence interval; E/O = ratio of expected to observed absolute risk; MBD = Mammographic Breast Density; MMHS = Mayo Mammography Health Study; PRS = Polygenic Risk Score; QRF = questionnaire-based risk factors

Table S7. Proportion of women reclassified at the high-risk thresholds based on risk estimated from the integrated model with questionnaire-based risk factors, 313-SNP PRS and density, as opposed to the model with questionnaire-based risk factors and 313-SNP PRS only

		US Population				SE Population			
		Total women		Future Cases		Total women		Future Cases	
QRF + PRS	QRF + PRS + MBD	n	%	n	%	n	%	n	%
AR<3%	AR<3%	34,043,694	77.9%	478,201	53.4%	1,095,322	87.6%	13,207	67.8%
AR≥3%	AR≥3%	6,230,381	14.3%	316,688	35.4%	88,032	7.0%	4,312	22.1%
AR<3%	AR≥3%	1,802,715	4.1%	62,325	7.0%	40,972	3.3%	1,404	7.2%
AR≥3%	AR<3%	1,641,370	3.8%	37,684	4.2%	25,369	2.0%	546	2.8%
AR<6%	AR<6%	42,135,743	96.4%	773,818	86.5%	1,230,034	98.4%	18,043	92.7%
AR≥6%	AR≥6%	822,237	1.9%	74,815	8.3%	8,118	0.65%	698	3.6%
AR<6%	AR≥6%	477,828	1.1%	32,986	3.7%	9,133	0.73%	611	3.1%
AR≥6%	AR<6%	282,353	0.6%	13,277	1.5%	2,410	0.2%	117	0.6%

Questionnaire-based risk factors correspond to the iCARE-Lit model and include age at menarche, age at first live birth, parity, oral contraceptive use, age at menopause, hormone replacement therapy use, type of hormone replacement therapy, alcohol intake, height, BMI, breast cancer family history (i.e., presence or absence of breast cancer in at least one first degree relative), benign breast disease. The 3% cutoff corresponds to the US Preventive Services Task Force recommendation for risk-lowering drugs [2] and the 6% cutoff corresponds to a cutoff for very high risk used in the WISDOM trial [3,4]. Abbreviations: AR = absolute risk, MBD = Mammographic Breast Density, PRS = polygenic risk score, SNP = single nucleotide polymorphism.

Table S8. Reclassification at high-risk thresholds after incorporating density to questionnaire-based risk factors and 313-SNP PRS.

	US Population				Sweden population			
	3% risk		6% risk		3% risk		6% risk	
	Total women, n (%)	Future cases, n (%)	Total women, n (%)	Future cases, n (%)	Total women, n (%)	Future cases, n (%)	Total women, n (%)	Future cases, n (%)
**Based on questionnaire-based risk factors + PRS**								
**Below threshold, n (%)**	35 846 408 (81.9)	540 525 (60.4)	42 613 571 (97.5)	806 804 (90.2)	1 136 294 (90.9)	14 611 (75.0)	1 239 167 (99.1)	18.654 (95.8)
**Above threshold, n (%)**	7 871 752 (18.1)	354 372 (39.6)	1 104 590 (2.5)	88 092 (9.8)	113 401 (9.1)	4 858 (25.0)	10 528 (0.9)	815 (4.2)
**Total, N**	43 718 160	894 897	43 718 161	894 896	1 249 695	19 469	1 249 695	19 469
**Based on questionnaire-based risk factors + PRS + MBD**								
**Reclassified, n (%)**	3 444 085 (7.9)	100 008 (11.2)	760 181 (1.7)	46 263 (5.2)	66 341 (5.3)	1 950 (10.0)	11 543 (0.9)	729 (3.7)
**Moving down, n (%)**	1 641 370 (3.8)	37 986 (4.2)	282 353 (0.6)	13 277 (1.5)	25 369 (2.0)	546 (2.8)	2 410 (0.2)	117 (0.6)
**Moving up, n (%)**	1 802 715 (4.1)	62 325 (7.0)	477 828 (1.1)	32 986 (3.7)	40 972 (3.3)	1 404 (7.2)	9 133 (0.7)	611 (3.1)

We report the number of women and future cases (i.e., women expected to develop breast cancer within 5 years) above or below the risk threshold based on questionnaire-based risk factors and PRS, and the number and percentage of these women moving below the threshold (down), above the threshold (up) and a total number and percentage of reclassified women. Questionnaire-based risk factors correspond to the iCARE- Lit model and includes age at menarche, age at first live birth, parity, oral-contraceptive use, age at menopause, hormone-replacement-therapy use, type of hormone replacement therapy, alcohol intake, height, BMI, breast-cancer family history (i.e., presence or absence of breast cancer in at least one first-degree relative), benign breast disease and the 313-SNP PRS. The 3% cut-off corresponds to the US Preventive Services Task Force recommendation for risk-lowering drugs [2] and the 6% cut-off corresponds to a cut-off for very high risk used in the WISDOM trial [3,4]. Abbreviations: MBD = Mammographic Breast Density, PRS = polygenic risk score, SNP = single nucleotide polymorphism.

Table S9: Overview of validation studies of breast cancer risk models including polygenic-risk-score and mammographic breast density

Model	Reference	Risk factors included	PRS	Breast density	Validation population	Sample size (cases/controls)
BCSC	Vachon 2015[5]	Age, race/ethnicity, family history, biopsy history	76-variant PRS	BI-RADS density	MMHS<br>MCBCS<br>BBCC	1643 / 2397
BOADICEA	Yang 2022[6]	Age, age at menarche, number of births, age at first live birth, OC, MHT, BMI, alcohol, smoking, personal BBD, family history of BC	313-variant PRS	BI-RADS density	KARMA	676 / 14826 (with PRS)<br>280 / 5413 (with PRS + PV)
IBIS/Tyrer-Cuzick	Van Veen 2018[7]	Family history, age, weight, height, parity, age at first child, menopausal status, age at menopause, age at menarche, BBD, LCIS, prior ATP, ovarian cancer	18-variant PRS	Percentage mammographic density	PROCAS	8897/ 466
	Brentnall 2020[8]	Family history, age, weight, height, parity, age at first child, menopausal status, age at menopause, age at menarche, BBD, LCIS, prior ATP, ovarian cancer	143-variant PRS	Percentage mammographic density	PROCAS	405 / 1668
iCARE-Lit	Current study	Age, age at menarche, parity, age at first birth, OC [a], HRT [b], type of HRT [b], age at menopause [b], BMI, height, alcohol, BBD, family history of breast cancer	313-variant PRS	BI-RADS density	MMHS<br>NHS I & II<br>KARMA	1468 / 19104

ATP: atypical hyperplasia, BBD: benign breast disease, BBCC: Bavarian Breast Cancer Cases and Control Study, BCSC: Breast Cancer Surveillance Consortium, iCARE: Individualized Coherent Absolute Risk Estimator, LCIS: lobular carcinoma in situ, MCBCS: Mayo Clinic Breast Cancer Study, MMHS: Mayo Mammography Health Study, MHT: Menopausal hormonal therapy, PRS: polygenic risk score, PV: pathogenic variants

[a] OC current use is only included in the iCARE Lit model for women younger than 50 years.

[b] Information on HRT and menopause is only included in the iCARE Lit model for women aged 50 years and older.

Figure S1A. Absolute risk and relative risk calibration in KARMA for women younger than 50 years

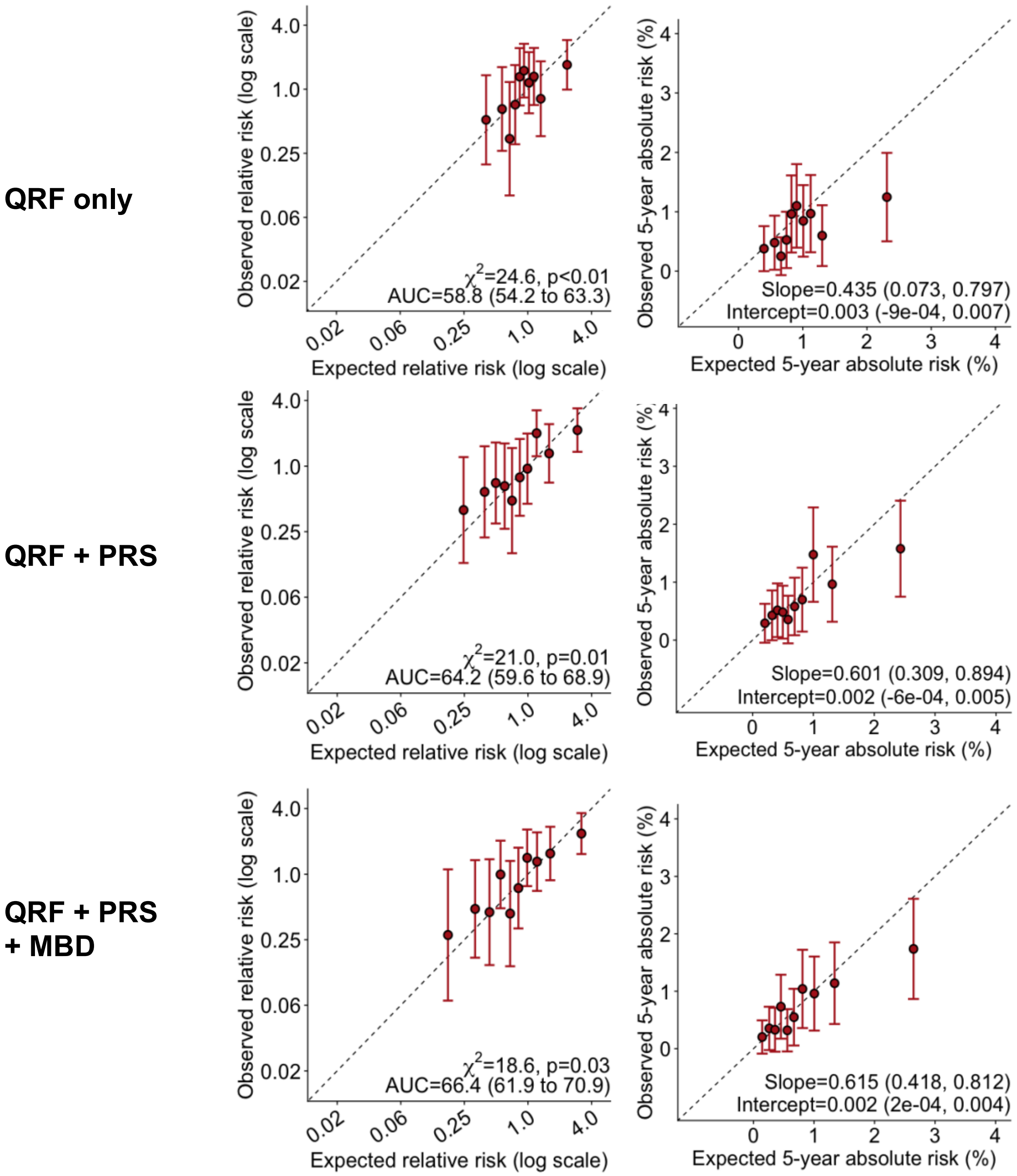

Calibration and discrimination of 5-year risk predictions of breast cancer for women aged younger than 50 years in the nested case-control sample of the Karolinska Mammography Project for Risk Prediction of Breast Cancer cohort with risk categories based on deciles of predicted 5-year absolute risk. Validation results are shown for the extended iCARE model that incorporates (i) questionnaire-based risk factors with a PRS based on 313 common germline variants without questionnaire-based risk factors, (ii) the fully integrated model incorporating questionnaire-based risk factors, the 313-variant PRS and BI-RADS breast density. Estimates and 95% CI of the calibration slope and intercept are reported based on a linear regression of the decile-specific observed proportion of cases within 5 years and the average of the predicted 5-year absolute risk. AUC = area under the curve, c^2 =chi-square goodness-of-fit test, CI = confidence interval, E/O = expected to observed number of cases, KARMA = Karolinska Mammography Project for Risk Prediction of Breast Cancer II, PRS = polygenic risk score, QRF = Questionnaire-based risk factors.

Figure S1B. Absolute risk and relative risk calibration in NHS II for women younger than 50 years

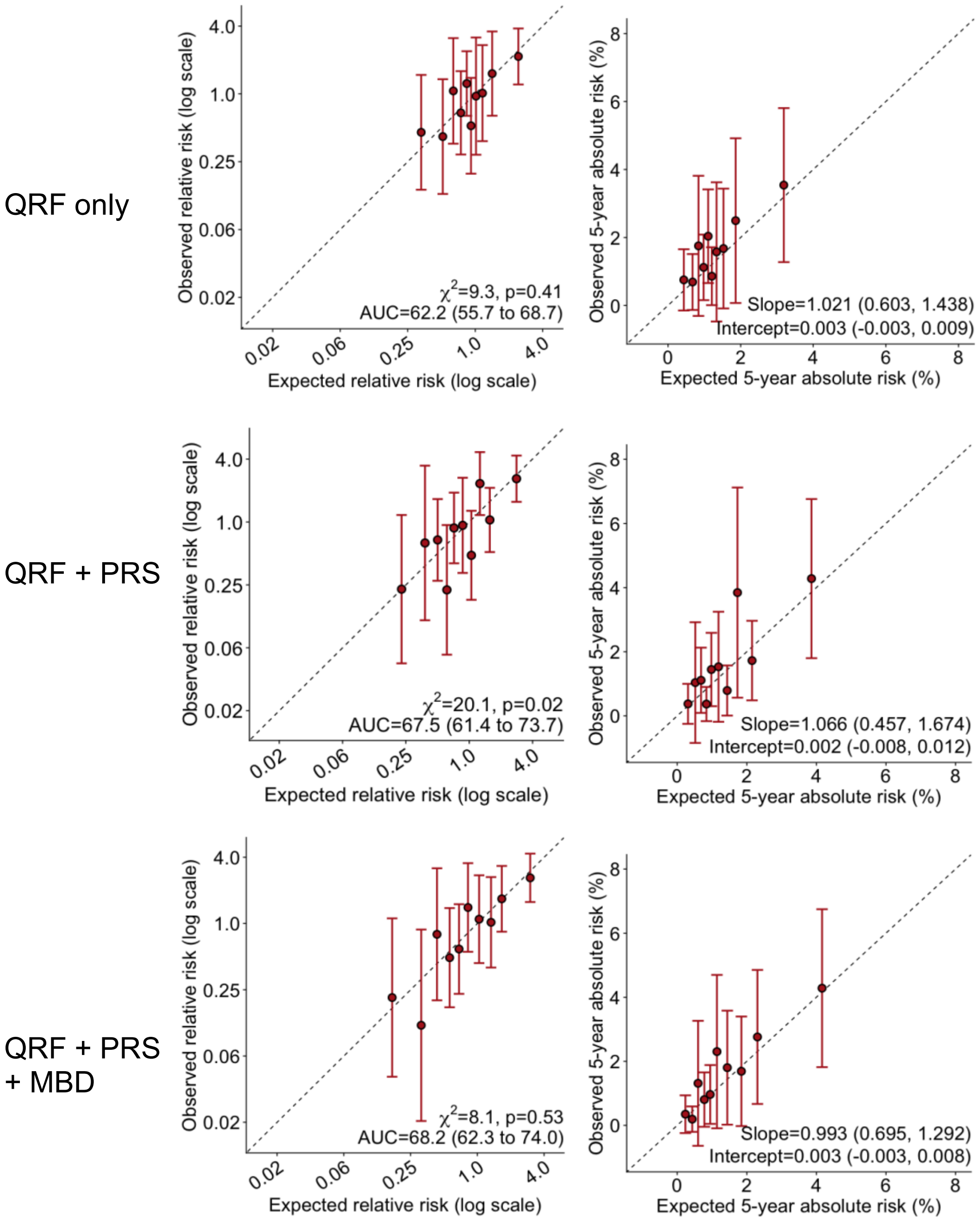

Calibration and discrimination of 5-year risk predictions of breast cancer for women aged younger than 50 years in the nested case-control sample of the Nurses' Health Study II with risk categories based on deciles of predicted 5-year absolute risk. Validation results are shown for the extended iCARE model that incorporates (i) questionnaire-based risk factors with a PRS based on 313 common germline variants without questionnaire-based risk factors, (ii) the fully integrated model incorporating questionnaire-based risk factors, the 313-variant PRS and BI-RADS breast density. Estimates and 95% CI of the calibration slope and intercept are reported based on a linear regression of the decile-specific observed proportion of cases within 5 years and the average of the predicted 5-year absolute risk. AUC = area under the curve, c^2 =chi-square goodness-of-fit test, CI = confidence interval, E/O = expected to observed number of cases, NHS II = Nurses' Health Study II, PRS = polygenic risk score, QRF = Questionnaire-based risk factors.

Figure S1C. Absolute risk and relative risk calibration in KARMA for women 50 years and older

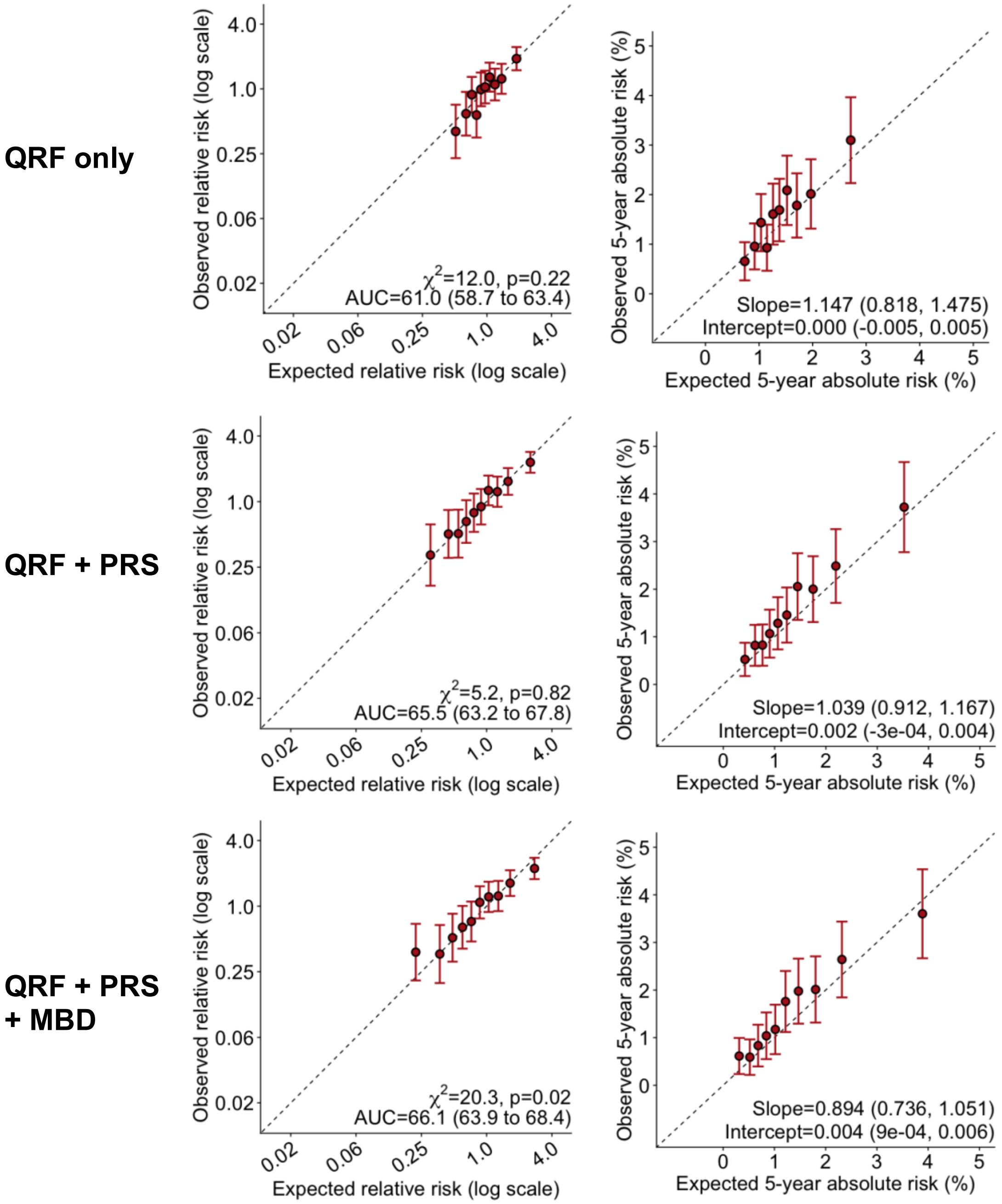

Calibration and discrimination of 5-year risk predictions of breast cancer for women aged 50 years and over in the nested case-control sample of the Karolinska Mammography Project for Risk Prediction of Breast Cancer cohort with risk categories based on deciles of predicted 5-year absolute risk. Validation results are shown for the extended iCARE model that incorporates (i) questionnaire-based risk factors with a PRS based on 313 common germline variants without questionnaire-based risk factors, (ii) the fully integrated model incorporating questionnaire-based risk factors, the 313-variant PRS and BI-RADS breast density. Estimates and 95% CI of the calibration slope and intercept are reported based on a linear regression of the decile-specific observed proportion of cases within 5 years and the average of the predicted 5-year absolute risk. AUC = area under the curve, c^2 =chi-square goodness-of-fit test, CI = confidence interval, E/O = expected to observed number of cases, KARMA = Karolinska Mammography Project for Risk Prediction of Breast Cancer, PRS = polygenic risk score, QRF = Questionnaire-based risk factors.

Figure S1D. Absolute risk and relative risk calibration in NHS I for women 50 years and older

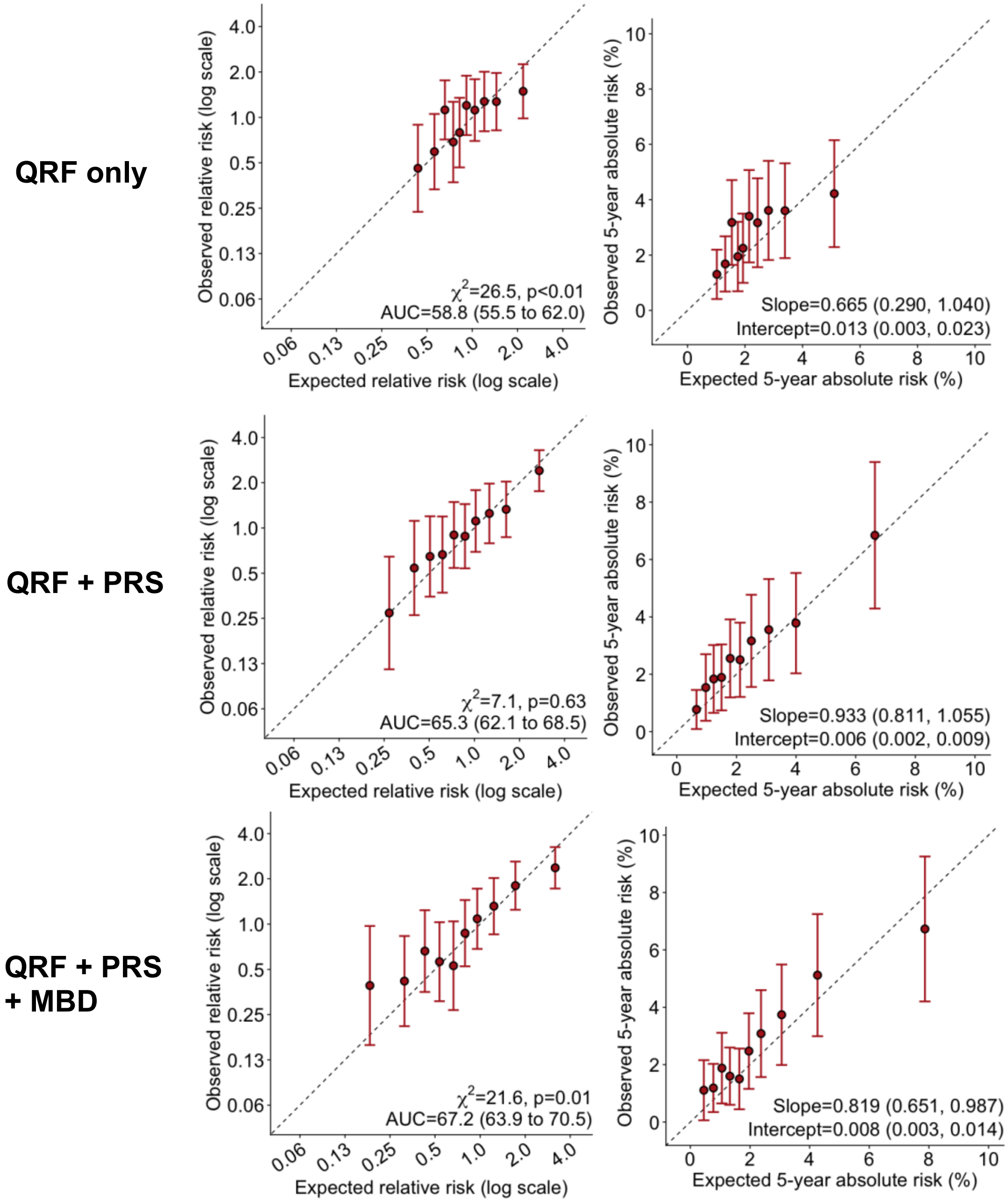

Calibration and discrimination of 5-year risk predictions of breast cancer for women aged 50 years and over in the nested case-control sample of the Nurses' Health Study I with risk categories based on deciles of predicted 5-year absolute risk. Validation results are shown for the extended iCARE model that incorporates (i) questionnaire-based risk factors with a PRS based on 313 common germline variants without questionnaire-based risk factors, (ii) the fully integrated model incorporating questionnaire-based risk factors, the 313-variant PRS and BI-RADS breast density. Estimates and 95% CI of the calibration slope and intercept are reported based on a linear regression of the decile-specific observed proportion of cases within 5 years and the average of the predicted 5-year absolute risk.
AUC = area under the curve, c^2 =chi-square goodness-of-fit test, CI = confidence interval, E/O = expected to observed number of cases, NHS II = Nurses' Health Study I, PRS = polygenic risk score, QRF = Questionnaire-based risk factors.

Figure S1E. Absolute risk and relative risk calibration in MMHS for women 50 years and older

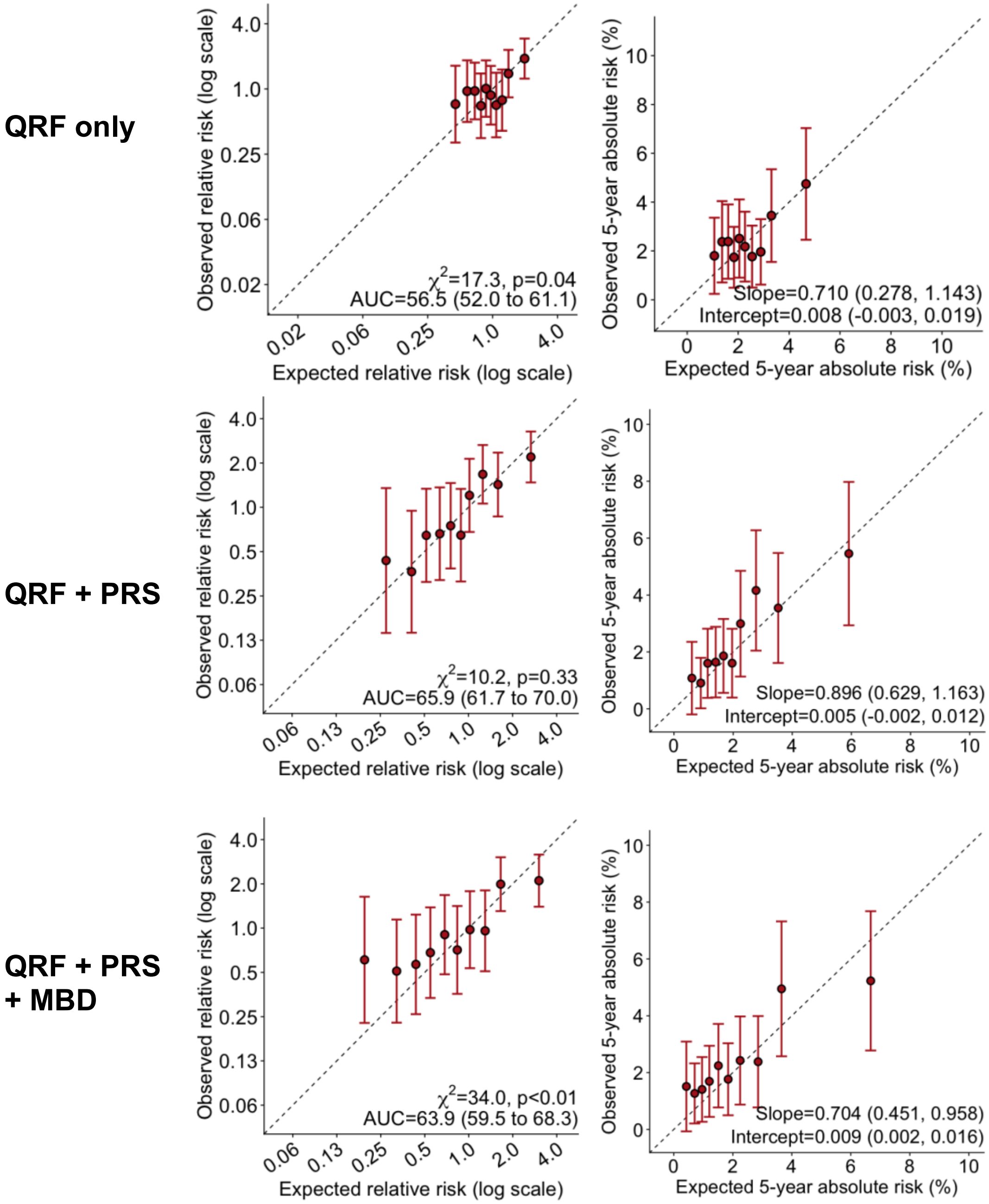

Calibration and discrimination of 5-year risk predictions of breast cancer for women aged 50 years and over in the nested case-control sample of the Mayo Mammography Health Study with risk categories based on deciles of predicted 5-year absolute risk. Validation results are shown for the extended iCARE model that incorporates (i) questionnaire-based risk factors with a PRS based on 313 common germline variants without questionnaire-based risk factors, (ii) the fully integrated model incorporating questionnaire-based risk factors, the 313-variant PRS and BI-RADS breast density. Estimates and 95% CI of the calibration slope and intercept are reported based on a linear regression of the decile-specific observed proportion of cases within 5 years and the average of the predicted 5-year absolute risk. AUC = area under the curve, c^2 =chi-square goodness-of-fit test, CI = confidence interval, E/O = expected to observed number of cases, MMHS = Mayo Mammography Health Study, PRS = polygenic risk score, QRF = Questionnaire-based risk factors.

References

1. Choudhury PP, Wilcox AN, Brook MN, Zhang Y, Ahearn T, Orr N, et al. Comparative validation of breast cancer risk prediction models and projections for future risk stratification. J Natl Cancer Inst. 2020;112(3):278–85.
2. Owens, D. K. *et al.* in *JAMA - Journal of the American Medical Association* Vol. 322 857-867 (American Medical Association, 2019).
3. Eklund, M. *et al.* The WISDOM Personalized Breast Cancer Screening Trial: Simulation Study to Assess Potential Bias and Analytic Approaches. *JNCI Cancer Spectrum*, doi:10.1093/jncics/pky067 (2019).
4. Shieh, Y. *et al.* Breast Cancer Screening in the Precision Medicine Era: Risk-Based Screening in a Population-Based Trial. *Journal of the National Cancer Institute*, doi:10.1093/jnci/djw290 (2017).
5. Vachon CM, Pankratz VS, Scott CG, Haeberle L, Ziv E, Jensen MR, et al. The contributions of breast density and common genetic variation to breast cancer risk. J Natl Cancer Inst [Internet]. 2015 [cited 2020 May 4];107(5). Available from: https://www.ncbi.nlm.nih.gov/pmc/articles/PMC4598340/
6. Yang X, Eriksson M, Czene K, Lee A, Leslie G, Lush M, et al. Prospective validation of the BOADICEA multifactorial breast cancer risk prediction model in a large prospective cohort study. J Med Genet. 2022;59(12):1196–205.
7. Van Veen EM, Brentnall AR, Byers H, Harkness EF, Astley SM, Sampson S, et al. Use of single-nucleotide polymorphisms and mammographic density plus classic risk factors for breast cancer risk prediction. JAMA Oncol. 2018 Apr 1;4(4):476–82.
8. Brentnall AR, Van Veen EM, Harkness EF, Rafiq S, Byers H, Astley SM, et al. A case-control evaluation of 143 single nucleotide polymorphisms for breast cancer risk stratification with classical factors and mammographic density. Cancer Epidemiol Int J Cancer [Internet]. 2020;146:2122–9. Available from: http://epi.grants.cancer.gov/oncoarray
